# Virological markers for clinical trials in chronic viral hepatitis

**DOI:** 10.1016/j.jhepr.2024.101214

**Published:** 2024-09-07

**Authors:** Jean-Michel Pawlotsky

**Affiliations:** 1 National Reference Center for Viral Hepatitis B, C and D, Department of Virology, Hôpital Henri Mondor (AP-HP), Université Paris-Est, Créteil, France; 2 Team “Viruses, Hepatology, Cancer”, Institut Mondor de Recherche Biomédicale, INSERM U955, Université Paris-Est, Créteil, France

**Keywords:** chronic hepatitis, virological markers, antiviral therapy, hepatitis B virus, hepatitis D virus, hepatitis C virus, hepatitis E virus, functional cure, partial cure, sustained virological response

## Abstract

Chronic hepatitis virus infections remain a major public health problem, despite significant therapeutic advances over the past two decades. Considerable progress has been made in the treatment of chronic viral hepatitis, but continued efforts are needed to develop and bring to market new drugs to fill the gaps in the current therapeutic armamentarium. Thus, clinical trials to assess the safety and efficacy of these new therapeutic approaches, including the selection of reliable and objective treatment endpoints, are still needed. Virological biomarkers play an important role in the diagnosis, monitoring, and evaluation of antiviral treatment efficacy. They are often used as primary or secondary endpoints in the evaluation of new treatments for chronic viral hepatitis. However, these markers are not all equally informative. The aim of this review article is to provide a comprehensive overview of the available virological tests for chronic viral hepatitis due to hepatitis B, D, C and E viruses, the information they provide and lack, the specific challenges associated with each, and their use in clinical trials of new treatments.

**Keypoints**

• Chronic hepatitis virus infections remain a major public health problem.
• Despite recent therapeutic successes, there is a need to continue efforts to develop and bring to market new drugs.
• Because of their correlation with the prognosis of liver disease, virological biomarkers are often used as primary or secondary endpoints in the evaluation of new treatments for chronic viral hepatitis.
• The “old” virological markers (HBV DNA, HBsAg, HBeAg/anti-HBe antibody) remain the cornerstone of assessing the efficacy of therapeutic approaches for HBV.
• Other virological markers (quantitative HBeAg, quantitative anti-HBc antibody, HBV RNA, HBcrAg, ultra-sensitive HBsAg and HBsAg isoforms) are recommended for exploratory and descriptive purposes only.
• Despite the lack of standardisation and incomparability of HDV RNA assays, HDV RNA quantification remains the primary biomarker of antiviral efficacy in HDV clinical trials.
• Sustained virological response, defined by undetectable HCV RNA or HCV core antigen at 12 or 24 weeks post-treatment, is the endpoint of anti-HCV therapy that indicates a definitive cure of the infection.
• Undetectable HEV RNA after the end of treatment is the endpoint of successful anti-HEV therapy, indicating a definitive cure of the infection.

## Introduction

Chronic hepatitis virus infections remain a major public health problem, despite significant therapeutic advances over the past two decades and efforts to reduce their incidence and prevent morbidity and mortality associated with cirrhosis and hepatocellular carcinoma. The World Health Organization (WHO) estimates the number of people with chronic hepatitis B virus (HBV) infection at approximately 254 million, with the virus still responsible for 1.1 million deaths per year, and an incidence of new HBV infections of approximately 1.2 million per year.^1^ Approximately 5% of patients with chronic HBV infection are also infected with hepatitis D virus (HDV), representing a global prevalence of approximately 13 million people.^2^ People with a double HBV-HDV infection have significantly more severe liver disease than those infected with HBV alone, and are at high risk of dying from their liver disease.^2^ The WHO also estimates that there are still approximately 50 million people living with chronic hepatitis C virus (HCV) infection worldwide, with HCV responsible for 242,000 deaths per year from complications of cirrhosis and/or hepatocellular carcinoma, and an incidence of approximately 1.0 million new HCV infections per year.^3^ Hepatitis E virus (HEV) infects approximately 20 million people each year. Infection is usually self-limiting and chronic HEV infection remains a rare event, affecting only immunocompromised patients, in whom it can lead to serious complications and in some cases death.^4^

Considerable progress has been made in the treatment of chronic viral hepatitis, which has had a very positive impact on the prognosis of these infections by preventing progression to cirrhosis, its complications and hepatocellular carcinoma. Lifelong use of nucleoside/nucleotide analogues (NUCs), including entecavir and tenofovir in both disoproxil fumarate and alafenamide forms, can reduce HBV replication to undetectable levels in most treated individuals, while time-limited use of pegylated interferon-α (PegIFNα) enables long-term control of infection in a small number of selected individuals.^5^ PegIFNα also reduces HDV replication during and sometimes after treatment, as does a new viral entry inhibitor, bulevirtide.^6^ The latest generation of pangenotypic direct-acting antivirals (DAAs), including sofosbuvir/velpatasvir, glecaprevir/pibrentasvir, sofosbuvir/daclatasvir, and sofosbuvir/velpatasvir/voxilaprevir, provide definitive cure of HCV infection in over 98% of cases,^7^ while ribavirin continues to be used to reduce replication and accelerate cure of chronic hepatitis E in immunocompromised patients.^8^ The main problems today remain the inadequate screening of chronic viral hepatitis worldwide, and the organisational and financial difficulties of access to care for infected persons, particularly in middle- and low-income countries.

Despite recent therapeutic successes, there is still a need to continue efforts to develop and bring to market new drugs to fill the gaps in current therapy, whether these are new regimens of limited duration for long-term control of HBV infection, antiviral treatments for effective long-term suppression of HDV replication until infection is cured, new HCV DAAs available for resource-limited countries, or specific antivirals for use against HEV infection. Thus, clinical trials to assess the safety and efficacy of these new therapeutic approaches, including the selection of reliable and objective treatment endpoints, are still needed.

The gold standard endpoint of any therapeutic development must be patient benefit in the context of addressing an unmet clinical need. However, it is often difficult to objectively demonstrate the reality of this benefit in the case of slowly progressive chronic diseases, where complications typically occur after many months or years. Therefore, it is necessary to identify biomarkers that predict the clinical benefit and to have them available to employ during the treatment phase of the trial or within a reasonable time after completion of the trial. In addition to having a strong predictive value for the clinical course and prognosis of the disease, these biomarkers must be standardised, automated if possible, FDA-approved, CE-marked and/or WHO-prequalified, and ideally, but not necessarily, commercially available. The best example is the use of HCV RNA testing in peripheral blood after treatment. Undetectable HCV RNA at 12 or 24 weeks after the end of antiviral treatment signals definitive cure of HCV infection and correlates with a very significant improvement in disease prognosis, including in patients with cirrhosis.^7^

Virological biomarkers play an important role in the diagnosis, monitoring, and evaluation of antiviral treatment efficacy. Because of their correlation with the prognosis of liver disease, they are often used as primary or secondary endpoints in the evaluation of new treatments for chronic viral hepatitis.[5], [6], [7], [8] However, these markers are not all equally informative. They must therefore be used with caution and with the utmost rigor in data interpretation. Expectations regarding the evaluated treatments must be carefully defined in order to understand the exact place of the tests and their significance, and to avoid their systematic use and a blinded description of their results, which do not necessarily answer the medical question initially posed.

The aim of this review article is to provide a comprehensive overview of the available virological tests for chronic viral hepatitis, the information they provide and lack, the specific challenges associated with each, and their use in clinical trials of new treatments.

## HBV

**Fig. 1:**
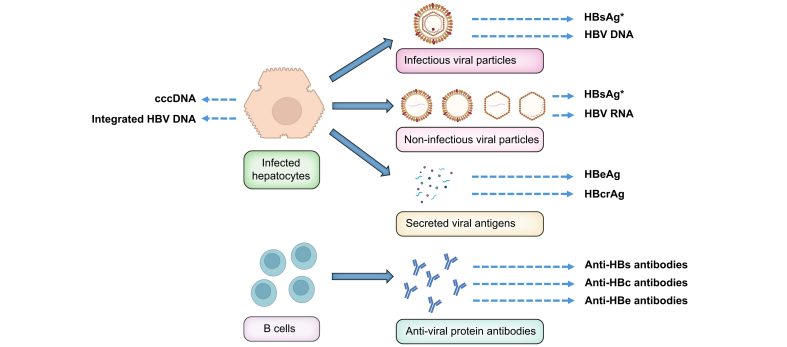
Origin of HBV virological markers. ∗Both cccDNA-derived and integrated sequence-derived HBsAg. cccDNA, covalently closed circular DNA; HBcrAg, hepatitis B core-related antigen; HBeAg, hepatitis B e antigen; HBsAg, hepatitis B surface antigen; HBV, hepatitis B virus.

A large number of therapeutic studies have been conducted in chronic hepatitis B, leading to the approval of NUCs and PegIFNα.^5^ These studies used virological endpoints: inhibition of viral replication measured by quantification of HBV DNA, loss of hepatitis B surface antigen (HBsAg) with or without anti-HBs antibody seroconversion, and HBe seroconversion, *i.e.* loss of hepatitis B e antigen (HBeAg) and appearance of anti-HBe antibodies in HBeAg-positive individuals. More recently, quantitative assays have emerged to measure the amount of HBsAg or HBe antigen in peripheral blood, in order to categorise and possibly stratify patients at enrolment and to assess the kinetics of these markers during treatment. So-called “new“ virological markers that are no longer new in 2024 have also been described, including the detection and quantification of HBV RNA and that of hepatitis B core-related antigen (HBcrAg) in peripheral blood, the clinical significance and utility of which remain unclear. Fig. 1 summarises the origin of the different HBV virological markers.

In the current context of the development of antiviral and immunomodulatory drugs aimed at the long-term control of HBV infection, while allowing for the discontinuation of treatment with NUCs,^9^ the detection/quantification of HBV DNA and of HBsAg remain essential as endpoints for the design of clinical efficacy trials, with the role of other markers remaining incidental, essentially for documentary purposes. However, in the context of the new therapies currently under investigation, the predictive value of HBV DNA (in patients with viral suppression by NUCs) and HBsAg remain to be demonstrated, in order to clarify their actual utility in relation to the unmet clinical needs of current treatment.

### HBV DNA

HBV DNA is produced intracellularly by reverse transcription of pregenomic RNA exclusively transcribed from covalently closed circular DNA (cccDNA) and then shed into the peripheral blood as a component of infectious virions (Fig. 1). HBV DNA is a robust marker of HBV replication that, in the absence of treatment, is strongly associated with the clinical prognosis of HBV-related liver disease, including disease progression, cirrhosis, complications of cirrhosis, hepatocellular carcinoma, and liver-related death.[10], [11], [12], [13]

In practice, HBV DNA is detected and quantified using automated molecular assays based on real-time PCR or transcription-mediated amplification (TMA). The lower limit of quantitation (LLOQ) of these tests is approximately 10 IU/ml. The LLOQ is the lowest value in the linear range of quantification, *i.e.* the lowest amount of HBV DNA that can be reliably quantified. Below the LLOQ, viral DNA may still be detectable, but the exact amount is very small and cannot be accurately determined. The DNA may also be undetectable (below the limit of detection [LOD], “target not detected”).

Commercial automated molecular HBV DNA tests are available in most virology laboratories in high-income countries. They may be more difficult to access in middle- and low-income countries. Whole blood samples can be collected on dried blood spots (DBS) for subsequent HBV DNA analysis in centralised laboratories, with excellent sensitivity and specificity.[14], [15], [16], [17], [18], [19] A meta-analysis of 12 studies from 4 continents showed that the sensitivity (ability to detect HBV DNA) of DBS was between 93% and 100% and the specificity was between 70% and 100% compared with tests performed directly on serum or plasma.^16^ However, the amount of HBV DNA measured on DBS is always 1.5 to 2 log lower than if the corresponding sample had been tested directly.^17^^,^^18^ Quantification of HBV DNA on DBS therefore has a lower limit of detection well above 10 IU/ml, varying between 900 and 4,000 IU/ml depending on the study.^16^

In practice, the presence of HBV DNA confirms the presence of viral replication and thus the diagnosis of active infection. It is interpreted in conjunction with blood alanine aminotransferase (ALT) levels and assessment of the stage of fibrosis by liver biopsy or, more commonly now, by a non-invasive biological or morphological test.^5^ The level of viral replication can be used to assess the prognosis of liver disease and to guide treatment decisions, although different international guidelines differ on the level of baseline HBV DNA that should trigger treatment. Baseline HBV DNA levels may be an important factor in selecting and stratifying patients for inclusion in therapeutic trials of antiviral and/or immunomodulatory drugs. The ability to reduce HBV DNA below the LLOQ (<10 IU/ml) is commonly used as a primary or secondary endpoint in HBV therapeutic trials. A reduction in viral load by a certain number of logs (usually 2 logs or more) without falling below 10 IU/ml may also be used in some cases.

Measurement of HBV DNA is an excellent marker of antiviral treatment efficacy in therapeutic trials, provided it is detectable and quantifiable prior to treatment initiation. In contrast, an effect on HBV replication cannot be monitored below the lower limit of detection of HBV DNA assays, which is a problem in patients already receiving NUC therapy, who represent the majority of patients enrolled in current therapeutic trials of antiviral and/or immunomodulatory approaches and who generally have undetectable HBV DNA. Whether further reductions in below-detectable HBV DNA levels improve the long-term prognosis of liver disease is unknown and needs to be investigated in long-term studies using more sensitive assays.

Ultra-sensitive HBV DNA assays, either qualitative only or qualitative and quantitative, exist but are not yet commercially available.^20^ The best way to use them is still under discussion. Ultra-sensitive measurement of HBV DNA levels may be useful to demonstrate the beneficial effect of compounds with anti-replication properties (*e.g.* capsid assembly modulators, nucleic acids, immune modulators affecting HBV DNA production) over several months to years of administration in combination with NUCs and/or other compounds under development.^21^

### HBs antigen/anti-HBs antibodies

HBsAg is carried by the three HBV proteins present on the surface of infectious viral particles and non-infectious spheres and filaments (Fig. 1). The long-term presence of HBsAg in the peripheral blood defines chronic HBV carriage and its different forms, recently redefined by EASL: HBeAg-positive chronic infection (immunotolerant); HBeAg-positive chronic hepatitis (immune reactive); HBeAg-negative chronic hepatitis; HBeAg-negative chronic infection (inactive carrier).^5^ HBsAg has long been considered as a reliable surrogate marker for the size of the cccDNA pool, the source of HBV persistence in the liver of infected patients. However, a substantial proportion, if not the majority, of the HBsAg-carrying protein transcription originates from integrated HBV DNA sequences, particularly in HBeAg-negative patients.^22^ Currently available HBsAg assays cannot distinguish between these two sources of production: viral transcription from cccDNA in the context of active replication *vs.* host genome transcription from integrated HBV sequences. It is unknown whether the balance between cccDNA- and integrated DNA-produced HBsAg influences the natural history of the disease.

HBsAg detection is based on automated, standardised ELISA assays with a lower detection limit of approximately 0.05 IU/ml, which varies slightly from assay to assay. Most of the current HBsAg assays are quantitative. Measuring the amount of HBsAg at baseline is a good predictor of loss of this marker during or after treatment, but the best predictor remains the dynamics of HBsAg levels during treatment: only patients with a significant decrease induced by therapy are likely to lose HBsAg at some point.[23], [24], [25], [26]

In resource-limited settings, HBsAg can be detected in blood collected on DBS and sent to a central laboratory, with overall sensitivities of 86-100% and specificities of 95-100% in a recent systematic review of a large number of international studies.^27^^,^^28^ Rapid diagnostic tests (RDTs) are also widely used to detect HBsAg and assess its loss in settings without access to automated ELISA.^27^ Their sensitivity has been shown to vary from RDT to RDT, and it is important to use only tests with high validated sensitivity compared to ELISA tests.

Ultra-sensitive ELISAs capable of detecting HBsAg down to 0.005 IU/ml have been developed.[29], [30], [31] The clinical utility of such sensitivity remains unknown, particularly as it reduces the likelihood of HBsAg loss during clinical trials. A research assay that measures HBsAg isoforms, including L-HBsAg and M-HBsAg, the large and intermediate products of the S gene, respectively, has provided no additional benefit over global quantification of HBsAg for staging disease or monitoring response to therapy.^32^

Various HBsAg endpoints have been defined for clinical trials, including: (i) HBsAg loss (<0.05 IU/ml); (ii) HBsAg <100 IU/ml; or (iii) a significant reduction in HBsAg levels from baseline. The ambitious EASL and AASLD-recommended composite primary endpoint for novel HBV therapies is “functional cure”, defined as HBsAg loss (<0.05 IU/ml) and HBV DNA below the LLOQ (<10 IU/ml) at 24 weeks off therapy.^33^ HBs seroconversion, characterised by the appearance of anti-HBs antibodies detected by ELISA, is a better guarantee that HBsAg loss will be maintained off therapy, but it is not required as an endpoint for new HBV therapies. A less demanding endpoint is “partial cure”, defined as HBsAg <100 IU/ml and HBV DNA below the LLOQ (<10 IU/ml) at 24 weeks off therapy.^33^

Loss of HBsAg is associated with improved clinical outcomes when it occurs spontaneously during the natural course of the disease and after PegIFNα treatment or after long-term NUC therapy.[34], [35], [36], [37] However, it has not been proven that HBsAg losses induced by new HBV drugs with different mechanisms of action (capsid assembly modulators, nucleic acids, immunomodulators, etc.) have the same impact on prognosis, especially in NUC-treated patients who already have a very significantly improved prognosis compared to untreated individuals.^21^ In other words, there may be several types of HBsAg losses depending on the treatment received, and their clinical impact may differ. This key question needs to be addressed in order to determine whether HBsAg loss is indeed required as a primary endpoint in clinical trials to demonstrate the clinical benefit of new HBV therapies under development.

### HBeAg/anti-HBe antibodies

HBeAg is carried by a viral protein that is shed by infected hepatocytes during viral replication (Fig. 1). During the course of chronic infection, the occurrence and selection of mutations in the pre-core region of the pre-C/C gene and/or in the basal core promoter region reduce or suppress HBeAg production, leading to HBe seroconversion (loss of HBeAg and appearance of anti-HBe antibodies). The presence or absence of HBeAg in the peripheral blood defines the stage of liver disease.^5^ The presence of HBeAg is associated with high levels of HBV replication. It may be used as a surrogate for HBV DNA when HBV DNA testing is not available.

HBeAg and anti-HBe antibodies are tested by automated commercial ELISAs, some of which are quantitative. HBeAg/anti-HBe antibody testing can also be performed with good sensitivity and specificity by a centralised laboratory using DBS specimens. RDTs are also available for HBeAg detection, although their sensitivity is slightly lower than that of automated ELISAs.^27^

HBeAg status, positive or negative, is an important parameter for inclusion or stratification of patients in therapeutic trials. HBe seroconversion is an important therapeutic endpoint in HBeAg-positive individuals enrolled in clinical trials, whether they have HBeAg-positive chronic hepatitis or are immunotolerant.^33^ HBe seroconversion can occur in two different clinical situations that need to be differentiated: (i) transition to the inactive carrier stage (chronic HBeAg-negative infection), characterised by undetectable or very low HBV DNA (<2,000 IU/ml), allowing treatment to be discontinued after consolidation (the risk of reactivation after treatment discontinuation requires regular monitoring for the presence of HBeAg and anti-HBe antibodies); or (ii) transition to HBeAg-negative chronic hepatitis characterised by high HBV DNA levels (>2,000 IU/ml) and fluctuating high serum ALT activity, which may warrant inclusion in new therapeutic trials.

### HBV RNA

Liver-produced HBV RNA is present in non-infectious virus-like particles, core protein-containing complexes and naked capsids in the peripheral blood of HBV-infected patients (Fig. 1).[38], [39], [40] The global level of circulating HBV RNA has been suggested as a potential surrogate marker of cccDNA transcriptional activity, presumably also reflecting the size of the cccDNA pool, but the latter has never been confirmed due to the limitations of cccDNA assays. It is unclear which form(s) of HBV RNA (pregenomic RNA and/or other viral transcripts, cccDNA-derived and/or integrated DNA-derived, spliced and/or unspliced) can be detected in peripheral blood. There are correlations between HBV RNA, HBV DNA and HBsAg levels in HBeAg-positive patients that are much weaker in HBeAg-negative patients.^41^^,^^42^ These relationships vary with HBV genotype.^43^ As recently reviewed, conflicting results have been reported regarding the ability of HBV RNA levels to predict the natural history of liver disease and virological outcomes during or after cessation of PegIFNα or NUC treatment in different groups of HBV-infected individuals (HBeAg-positive *vs.* -negative, different genotypes, *etc.*).^43^ The decrease in HBV RNA levels on NUCs is a predictor of subsequent HBeAg seroconversion, and high HBV RNA levels are also associated with a risk of reactivation and ALT flare after NUC cessation in patients with undetectable HBV DNA.^43^

There are no commercially available assays for HBV RNA. Several in-house methods can be used to detect and quantify HBV RNA, including classical reverse-transcription quantitative PCR (RT-qPCR), 3’ rapid amplification of cDNA ends PCR, or digital droplet PCR. In 2024, HBV RNA assays are not standardised and have not been adequately compared, so their analytical and clinical performance is largely unknown. They do not provide information on the composition of the RNA mixture they detect and quantify, nor its changes during and after different therapies.

Despite the lack of standardisation of available assays, HBV RNA quantification is now included in most clinical trials in HBV-infected patients. The primary value of measuring HBV RNA level appears to be its use as a marker of “target engagement” for new drugs that directly or indirectly block HBV RNA transcript production, whether derived from cccDNA and/or integrated HBV sequences (*e.g*., silencing RNAs, antisense oligonucleotides or nucleic acid polymers). However, careful examination of individual HBV RNA, HBsAg and HBV DNA responses to these compounds in phase II clinical trials suggests more complicated mechanisms than the simple blockade of transcript production translating into HBsAg level reduction, and a complex role of other compounds used in combination, such as PegIFNα and/or NUCs.[44], [45], [46], [47], [48], [49] Correlations with subsequent clinical outcomes and potential clinical benefit to patients have not been established.^50^

Overall, HBsAg remains the appropriate endpoint of response for drugs targeting transcript production, and HBV RNA cannot be used as a credible primary or secondary endpoint for new HBV therapies in development. Because its utility is elusive, EASL and AASLD recommend that HBV RNA quantification be considered as an exploratory tool to provide additional information in future clinical trials, provided that the method used and primer selection are specified.^33^

### HBcrAg

HBcrAg is a composite biomarker composed of three antigenic components that are shed into the general circulation in currently unknown proportions: denatured HBeAg (which predominates), the capsid antigen HBcAg, and the precore-related antigen p22 (a precursor of HBeAg) (Fig. 1).^43^ The existence of HBcrAg has been debated, and some have suggested that it is predominantly, if not exclusively, HBeAg,^51^ making it of little use in HBeAg-negative individuals. Significant relationships have been reported with HBV DNA and HBsAg levels, but the scatter of the points is large and these relationships are likely to reflect that which exists between HBeAg and the level of viral replication in HBeAg-positive patients.^52^

HBcrAg has been suggested as a potential indirect marker of cccDNA transcriptional activity and cccDNA pool size. However, the relationship between HBcrAg levels, the amount of cccDNA in liver biopsies and the level of transcriptional activity of this cccDNA was observed mainly in HBeAg-positive individuals, again suggesting a predominant role for the HBeAg component.^53^ HBcrAg levels discriminate well between HBeAg-positive and HBeAg-negative individuals, but less well between chronic hepatitis and chronic infections within each of these two groups.^54^ There also appears to be an association between HBcrAg titres and the risk of hepatocellular carcinoma, probably related to the existing association with HBV DNA levels.^55^ As with HBV RNA, the persistence of HBcrAg in the absence of DNA on treatment is predictive of relapse when NUC therapy is discontinued.^56^

HBcrAg levels can be measured by a commercial, non-FDA-approved, non-CE-marked chemiluminescent enzyme immunoassay. The linear range of quantification of the test is 1,000 to 7,500,000 U/ml (3.0 to 6.9 log U/ml). HBcrAg levels can be measured on DBS blood, with a loss of sensitivity compared to direct peripheral blood detection.^57^ Recently, an ultra-sensitive immunoassay with a LLOQ of 125 U/ml (2.1 log U/ml) was developed.^58^^,^^59^

The problem of measuring HBcrAg levels in clinical trials in patients with hepatitis B is similar to that of quantifying HBV RNA. The test exists and is generally performed during these trials, but the significance of the changes observed remains difficult to interpret, as they appear to essentially reflect changes in the amount of HBeAg.[44], [45], [46], [47], [48], [49] Correlations with subsequent clinical outcomes and potential clinical benefit to patients have not been established. Overall, HBcrAg, like HBV RNA, cannot be used as a credible primary or secondary endpoint for new HBV therapies in development. EASL and AASLD recommend its use as an exploratory tool to provide additional information in future clinical trials.^33^

### Quantitative anti-HBc antibodies

Anti-HBc antibodies can be quantified in peripheral blood by enzyme immunoassays. However, the dynamic range of quantification of these assays is narrow, and many methods are only semi-quantitative. Anti-HBc antibody levels correlate with stages of infection and severity of liver disease. They are associated with the likelihood of HBeAg and HBsAg seroclearance, but have a low individual predictive value for treatment outcome with old and new therapies in development and can only be used as an exploratory tool to provide additional information in future clinical trials.^33^^,^^60^

### HBV genotype

HBV has 10 genotypes (A to J) and over 40 subgenotypes based on the divergence of ≥8% and 4 to <8% in the complete genome, respectively.^61^ HBV genotype can be determined by sequencing a portion of the viral genome using population sequencing or next-generation sequencing (NGS), followed by phylogenetic analysis against a complete sequence database. HBV genotype must be systematically determined at the time of enrolment in therapeutic trials as it can be used for stratification purposes and knowledge of it is essential for interpretation of results.^33^ Viruses with different genotypes may respond very differently to the different therapeutic approaches being tested.

### HBV resistance

Regardless of the agents administered, the presence of HBV DNA detectable on treatment and in sufficient quantity to permit sequencing of the viral genome (3 log IU/ml or greater) should prompt a search for resistance-associated substitutions (RASs) that may explain the persistence of viral replication. This includes population sequencing or NGS of the target region(s) of the administered treatment, *i.e.* reverse transcriptase for NUCs, core for capsid assembly modulators, and/or nucleic acid target region(s), in search of amino acid substitutions that confer reduced susceptibility to these drugs.^33^ This determination should be made prior to treatment initiation if DNA is detectable (patients not treated with NUCs) and at the time of post-treatment escape or relapse. There are two possible scenarios: (i) the selection of known RASs is observed; (ii) amino acid changes are observed in one or more target regions of the administered products that are not known to confer reduced sensitivity to the administered compounds. In this case, the effect of these substitutions on the susceptibility of the corresponding viruses must be characterised *in vitro* in appropriate models by a specialised laboratory.

### cccDNA and integrated HBV DNA sequences

Measuring the size of the cccDNA pool in the liver and the effect of treatments on it is the ideal primary endpoint for any therapeutic approach aimed at reducing or even eliminating this pool. The ability to measure the proportion of viral DNA present in the form of cccDNA and that integrated into host genomes, as well as their respective transcriptional activities, would also be an ideal therapeutic endpoint. Unfortunately, this has not been possible to date for two reasons. The first is the accessibility of the study material. Except in the very exceptional case of proof-of-concept studies, it is not possible to perform iterative liver biopsies in clinical trials for ethical and patient acceptance reasons. Fine needle aspiration is a less invasive alternative to liver biopsy.^62^ However, this approach appears to favour aspiration of mononuclear cells from the microenvironment, particularly blood, to the detriment of hepatocytes, and this needs to be validated. The second reason is the lack of standardisation of cccDNA and integrated sequence detection and quantification techniques, which have all been developed independently by research laboratories and whose rare comparisons show very significant variations in performance, casting doubt on the veracity of the results obtained.^63^ Standardisation of technologies for quantification of cccDNA and integrated viral sequences on liver tissue is now a priority.

### HBV summary

In summary, there is a consensus today that the “old” virological markers remain the cornerstone for assessing the efficacy of the various therapeutic approaches under investigation in patients with chronic HBV infection (Table 1). Detection/quantification of HBV DNA and HBsAg, as well as HBeAg/anti-HBe antibody complex testing, must be performed systematically before, during, at the end of treatment, and in the long-term off-treatment period to assess response and its durability (Table 1). Several primary or secondary endpoints can be defined with these markers: (i) a significant (>1 log) reduction in viral load or the achievement of undetectable HBV DNA (<10 IU/ml) during treatment; (ii) HBe seroconversion in HBeAg-positive patients; (iii) a reduction in HBsAg level <100 IU/ml with undetectable HBV DNA (<10 IU/ml) at 24 weeks post-treatment, defining “partial cure”; (iv) undetectable HBsAg (<0.05 IU/ml) in combination with undetectable HBV DNA (<10 IU/ml) at 24 weeks post-treatment, defining “functional cure”.^33^ The appearance of anti-HBs antibodies should be monitored in patients who have achieved functional cure. These virological endpoints must be interpreted in the context of evaluating the clinical impact of treatment in the short, medium and long term, using serum ALT levels, changes in non-invasive markers of liver fibrosis, clinical events, and quality-of-life indices.

**Table 1:** HBV virological markers to assess the efficacy of anti-HBV drugs in clinical trials.

Virological marker	Type of assay	Timing of assessments	Endpoints (primary and secondary)
HBV genotype	Population sequencing or NGS	Baseline	• Interpretive
Quantitative HBV DNA	Real-time quantitative PCR or TMA	Baseline On treatment End of treatment 24 weeks off-treatment Long-term off-treatment	• Significant reduction (>1 log) in HBV DNA level • Undetectable HBV DNA (<10 IU/ml) • HBe seroconversion • “Partial cure”: reduction in HBsAg level <100 IU/ml with undetectable HBV DNA (<10 IU/ml) at 24 weeks post-treatment • “Functional cure”: undetectable HBsAg (<0.05 IU/ml) combined with undetectable HBV DNA (<10 IU/ml) at 24 weeks post-treatment (with or without anti-HBs seroconversion)
Quantitative HBsAg (& anti-HBs antibodies)	Enzyme immunoassay	Baseline On treatment End of treatment 24 weeks off-treatment Long-term off-treatment	
HBeAg/anti-HBe antibodies	Enzyme immunoassay	Baseline On treatment End of treatment 24 weeks off-treatment Long-term off-treatment	
Quantitative HBV RNA	Real-time reverse-transcription PCR, 3’ RACE PCR, or digital droplet PCR	Baseline On treatment End of treatment 24 weeks off-treatment Long-term off-treatment	• Optional, exploratory, descriptive
Quantitative HBcrAg	Chemiluminescent enzyme immunoassay	Baseline On treatment End of treatment 24 weeks off-treatment Long-term off-treatment	
Quantitative anti-HBc antibody	Enzyme immunoassay	Controversial	
HBV resistance	Population sequencing or NGS∗	Baseline Treatment failure (breakthrough or relapse)	• RAS selection
cccDNA and integrated HBV DNA sequences	In-house molecular methods	Controversial	• Optional, basic research if liver biopsy available

3’ RACE, 3’ rapid amplification of cDNA ends; cccDNA, covalently closed circular DNA; HBcrAg, hepatitis B core-related antigen; HBeAg, hepatitis B e antigen; HBsAg, hepatitis B surface antigen; HBV, hepatitis B virus; TMA, transcription-mediated amplification.

∗ Viral genome detectable on treatment and in sufficient quantity to permit sequencing.

The so-called “new” virological markers are no longer new and are yet to demonstrate clinical utility for the evaluation of treatments for HBV infection. These tests, including quantitative HBeAg, quantitative anti-HBc antibody, HBV RNA, HBcrAg, ultra-sensitive HBsAg and HBsAg isoforms, are recommended for exploratory and descriptive purposes when available (Table 1).^33^ A new generation of virological markers with strong predictive value for clinical outcomes would be welcome.

## HDV

For many years, PegIFNα has been the only available treatment for chronic HDV infection, although it is not formally approved for this indication. Bulevirtide, an HDV entry inhibitor, recently received conditional approval from the European Medicines Agency and other compounds are being evaluated, alone or in combination, in clinical trials.^6^ The primary endpoint of HDV treatment is the effect of therapy on HDV RNA levels, which measure the level of viral replication. However, since HDV infection occurs only in chronic HBV carriers, HBV virological markers also play an important role in the evaluation of treatments for hepatitis D.

### HDV RNA

HDV RNA is present in viral particles as single-stranded RNA organised into an unbranched rod-like structure by extensive molecular base pairing. HDV RNA replication is catalysed by nuclear hepatocyte RNA polymerases via a double rolling circle mechanism.^64^ Genomic HDV RNA is targeted to the cytoplasm for assembly into ribonucleoproteins. HDV infection is productive in HDV-infected cells that are also infected with HBV, leading to release of HDV RNA into viral particles in the peripheral blood. In hepatocytes that are not infected with HBV, HDV infection is abortive. However, HDV RNA can survive in HBV-uninfected cells for at least 6 weeks before HDV replication is rescued by subsequent HBV infection.^64^

The amount of HDV RNA in peripheral blood is a direct marker of HDV replication in the liver. There is an association between the presence of HDV RNA and the occurrence of liver complications in HBV-HDV-infected patients, including cirrhosis, decompensation of cirrhosis and hepatocellular carcinoma.[65], [66], [67] However, it is unclear whether HDV RNA levels correlate with the incidence of these complications, in part because of the variability and incomparability of assays between studies.

HDV RNA is detected and quantified in peripheral blood using nucleic acid assays based on real-time reverse-transcription PCR. The unique structure of HDV RNA (variability in primary sequences, secondary structures, circular nature), which affects both the extraction, reverse transcription and PCR amplification steps, poses several challenges for HDV RNA quantification in clinical practice. Many assays used in clinical trials have been developed in-house. Commercial assays have also been developed, some of which are CE marked. As recently reviewed,^68^ several factors are responsible for the considerable variability in performance of HDV RNA assays, including the RNA extraction method, primer/probe design, lack of automation and lack of standardisation between laboratories, despite the availability of a WHO standard.^69^ Due to the lack of standardisation, variability in analytical sensitivity and inconsistency in quantification between assays, it is not possible to define a reliable LOD or LLOQ. As a result, changes in HDV RNA levels have more value in clinical studies than absolute levels in IU/ml. Whole blood samples may also be collected on DBS for subsequent HDV RNA analysis by centralised laboratories, with the same assay limitations and a reduced analytical sensitivity.^15^

In practice, the presence of HDV RNA confirms the presence of viral replication and thus the diagnosis of active HDV infection. The presence of HDV RNA is the key factor in selecting patients for inclusion in therapeutic trials and in assessing response to antiviral therapy.^33^ The preferred endpoint of successful HDV therapy is HBsAg loss, with or without anti-HBs seroconversion, together with HDV RNA below the LLOQ. However, if HBsAg loss cannot be achieved, HDV RNA below the LLOQ on or after treatment is the preferred endpoint of therapy. A reduction in HDV RNA levels of ≥2 log IU/ml has been used as an acceptable endpoint in phase II and III clinical trials of anti-HDV drugs, both on and off therapy.[70], [71], [72] A log decrease of less than 1 log IU/ml defines a virological non-response. Some studies have used a “combined endpoint”, *i.e.* the combination of a greater than 2 log reduction in HDV RNA level and normalisation of ALT.^70^^,^^71^^,^^73^ This combined endpoint is confusing because it evaluates two parameters that are different in nature, although related, and should be interpreted independently.

### HDV genotype

HDV has eight genotypes (HDV-1 to HDV-8) and several subgenotypes.^74^ HDV genotype can be determined by sequencing a portion of the viral genome using population sequencing or NGS, followed by phylogenetic analysis against a complete sequence database (in-house methods). HDV genotype must be determined at the time of enrolment in therapeutic trials, as knowledge of it is essential for interpretation of results and possible inconsistencies in HDV RNA quantification with the least common genotypes.^33^

### HDV/HBV resistance

The presence of HDV RNA detectable in sufficient quantities to allow for sequencing of the viral genome during or after antiviral treatment that directly targets an HDV function should prompt a search for RASs that may explain the persistence of viral replication. This includes population sequencing or NGS of the target region(s) of the administered antivirals to search for nucleotide or amino acid substitutions that confer reduced susceptibility to these drugs. This should be done at the start of treatment and at the time of escape or relapse.^33^ In patients receiving therapies that target the HBV cycle (*e.g*., the HBV entry inhibitor bulevirtide), the HBV target region of the drug(s) should also be sequenced in the search for HBV resistance to the compound(s).^75^ If amino acid changes are observed in one or more HDV or HBV target regions of the administered products that are not known to confer reduced susceptibility to the administered compounds, the effect of these substitutions on the susceptibility of the corresponding viruses must be characterised *in vitro* in appropriate models by a specialised laboratory.

### HDV summary

Despite the lack of standardisation and incomparability of HDV RNA assays, HDV RNA quantification remains the primary biomarker of antiviral efficacy in clinical trials enrolling patients with HDV infection (Table 2). HDV genotyping and, ideally, HBV genotyping should be performed at treatment baseline in all clinical trials. There is consensus on the recommendation to perform HDV RNA quantification, HBV DNA quantification, HBeAg/anti-HBe antibody detection and HBsAg detection/quantification at baseline, during treatment, at the end of treatment and 24 weeks after the end of treatment (Table 2).^33^ Multiple endpoints can be defined with these markers for both finite and maintenance therapeutic strategies.

**Table 2:** HDV virological markers to assess the efficacy of anti-HDV drugs in clinical trials.

Virological marker	Type of assay	Timing of assessments	Endpoints (primary and secondary)
HDV genotype	Population sequencing or NGS	Baseline	• Interpretive
Quantitative HDV RNA	Real-time reverse transcriptase PCR	Baseline On treatment End of treatment 24 weeks off-treatment Long-term off-treatment	• ≥2 log reduction in HDV RNA levels at 48 weeks of treatment (maintenance strategies) • HDV RNA below the LLOQ at 24 weeks of treatment (maintenance strategies) • HDV RNA below the LLOQ at 48 weeks of treatment and maintained over time post-treatment (finite treatment) • HDV RNA below the LLOQ and HBsAg loss with or without anti-HBs seroconversion maintained over time post-treatment (finite treatment)
Quantitative HBsAg	Enzyme immunoassay	Baseline On treatment End of treatment 24 weeks off-treatment Long-term off-treatment	
HDV resistance	Population sequencing or NGS∗	Baseline Treatment failure (breakthrough or relapse)	• RAS selection
HBV resistance	Population sequencing or NGS∗	Baseline Treatment failure (breakthrough or relapse)	• RAS selection (drugs targeting HBV)

HBsAg, hepatitis B surface antigen; HBV, hepatitis B virus; LLOQ, lower limit of quantitation; NGS, next-generation sequencing; RAS, resistance-associated substitution.

∗ Viral genome detectable on treatment and in sufficient quantity to permit sequencing.

The preferred endpoint for finite anti-HDV therapeutic strategies is HDV RNA below the LLOQ of the assay (no absolute level defined) and HBsAg loss with or without anti-HBs seroconversion maintained after treatment. However, if HBsAg loss is not achieved, HDV RNA below the LLOQ at 24 weeks post-treatment is an acceptable endpoint, provided that at least 5 years of follow-up is performed. For maintenance strategies, the preferred endpoint is HDV RNA below the LLOQ at 48 weeks of treatment and maintained over time (Table 2). However, these ambitious endpoints have rarely been achieved, leading investigators to set a less ambitious endpoint of a ≥2 log reduction in HDV RNA levels at 48 weeks of treatment. The use of a “combined endpoint” linking an HDV RNA level reduction of ≥2 log IU/ml and ALT normalisation at 48 weeks of treatment was confusing because ALT changes can be related to a variety of often complex events, including the antiviral effect of the compounds or their anti-inflammatory activity (both of which can reduce ALT levels), but also ALT flares related to the antiviral response and drug-related liver toxicity (both of which can increase ALT levels). Therefore, the use of purely virological HDV and HBV endpoints remains preferable for future clinical trials. These virological endpoints need to be interpreted in the context of assessing the clinical impact of treatment in the short, medium and long term, using serum ALT levels, changes in non-invasive markers of liver fibrosis, clinical events and quality-of-life indices.

Other tests, including quantitative HBeAg, quantitative anti-HBc antibody, HBV RNA, HBcrAg, ultra-sensitive HBsAg and HBsAg isoforms, intrahepatic HDV RNA and HDV antigen, should be limited to exploratory and descriptive purposes when available.^33^

## HCV

Pangenotypic DAA regimens have changed the landscape of HCV therapy.^7^ Today, sofosbuvir/daclatasvir, sofosbuvir/velpatasvir, glecaprevir/pibrentasvir and sofosbuvir/velpatasvir/voxilaprevir, used as first-line or retreatment regimens, achieve definitive viral clearance in more than 98% of cases. One might assume that no further clinical trials are needed. However, not all of these combinations are available as low-cost generics, and even those are not available everywhere in the world and are sometimes too expensive, limiting options for HCV-infected patients. As a result, clinical trials of new DAA combinations are still ongoing in low- and middle-income countries.

### HCV RNA

HCV RNA is present in viral particles as single-stranded positive-sense RNA. Replication occurs in the cytoplasm of infected cells and is catalysed by a viral RNA-dependent RNA polymerase that synthesises negative-sense replication intermediates that serve as templates for the synthesis of genomic RNA, which is also used as mRNA for viral protein synthesis. The amount of HCV RNA in peripheral blood is a direct marker of HCV replication in the liver and a strong predictor of liver disease progression and complications, including cirrhosis, decompensation of cirrhosis, and hepatocellular carcinoma.^7^

HCV RNA is detected and quantified in peripheral blood using quantitative nucleic acid assays based on real-time reverse-transcription PCR or transcription-mediated amplification. Several commercial assays are available that are well standardised to the WHO standard, sensitive with a LOD/LLOQ around 10-20 IU/ml, and highly specific. Whole blood samples can also be collected on DBS for subsequent HCV RNA analysis by centralised laboratories, with a loss of analytical sensitivity of approximately 1.5 to 2 log.^27^^,^^76^

In practice, the presence of HCV RNA confirms the diagnosis of active HCV infection. It is the key factor in selecting patients for inclusion in therapeutic trials and in assessing response to antiviral therapy. The endpoint of successful HCV therapy is sustained virological response (SVR), defined as undetectable HCV RNA at 12 weeks (SVR12) or 24 weeks (SVR24) post-therapy.^7^ SVR indicates definitive cure of the infection.

### HCV core antigen

HCV core antigen is produced by hepatocytes during the life cycle of HCV and shed into the general circulation as the major component of viral capsids. It can be detected and quantified in plasma or serum by commercially available standardised enzyme immunoassays. HCV core antigen levels correlate closely with HCV RNA levels, but core antigen assays are less sensitive than HCV RNA assays (LOD equivalent to 500 to 3,000 IU/ml, depending on HCV genotype).[77], [78], [79], [80] HCV core antigen assays are not sensitive enough to be used with whole blood collected on DBS.

The presence of HCV core antigen is a marker of active viral replication, and core antigen testing can be used as a surrogate for HCV RNA to determine SVR at 12 or 24 weeks post-therapy.^7^ Thus, undetectable HCV core antigen post-treatment may be used as an endpoint in HCV clinical trials.

### HCV genotype

To date, eight HCV genotypes (1 through 8) and over 100 subtypes have been reported, and more may exist. The HCV genotypes differ from each other by approximately 30%-35% and the subtypes differ by >15% of their nucleotide sequence.^81^ HCV genotyping assays are based on either reverse hybridisation of PCR products targeting a specific region of the genome (5’ non-coding region, core region) or sequencing, including population sequencing or NGS, of the NS5B region or, less commonly, other genomic regions, followed by phylogenetic analysis. The HCV genotype must be systematically determined at the time of enrolment in therapeutic trials. It can be used for stratification purposes and knowledge of it is essential for interpretation of results. In clinical trials, sequencing (of the NS5B or another region) should be preferred, because it allows for subtype determination, a particularly useful piece of information in the context of the increasing circulation of “unusual“ HCV subtypes that may be inherently resistant to some DAAs, characterised by lower SVR rates to certain DAA combinations than those reported with the most common HCV subtypes in phase III clinical trials.^81^

### HCV resistance

Population sequencing or NGS of the target region(s) of the administered DAAs in search of RASs (amino acid substitutions conferring reduced susceptibility to these drugs) must be performed in all cases of treatment failure, *i.e.* in the absence of SVR, in comparison with baseline sequences.^82^ This determination will reveal the presence at baseline of polymorphisms at known RAS positions, the selection of HCV RASs by therapy, or amino acid changes in the target regions of the administered products that are not yet known to confer reduced susceptibility to the administered compounds. In this case, the effect of these substitutions on the susceptibility of the corresponding viruses must be characterised *in vitro*.

### HCV summary

Despite the availability of highly effective pangenotypic DAA combinations, clinical trials are still ongoing, particularly in low- and middle-income countries, to bring new combinations to the market. HCV genotype and subtype must be determined prior to treatment using methods suitable for accurate subtype determination, as well as the sequence of the target regions of the administered DAAs (Table 3). SVR, the endpoint of therapy, must be assessed at 12 or 24 weeks post-treatment using HCV RNA or HCV core antigen testing. In the case of treatment failure, the DAA target regions must be sequenced at the time of relapse to search for selected RASs (Table 3).

**Table 3:** HCV virological markers to assess the efficacy of anti-HCV drugs in clinical trials.

Virological marker	Type of assay	Timing of assessments	Endpoints (primary and secondary)
HCV genotype	Population sequencing or NGS	Baseline	• Interpretive
Quantitative HCV RNA	Real-time reverse transcriptase PCR or TMA	Baseline 12 or 24 weeks post-treatment	• SVR12 or SVR24
Quantitative HCV core antigen	Enzyme immunoassay	Baseline 12 or 24 weeks post-treatment	• SVR12 or SVR24
HCV resistance	Population sequencing or NGS∗	Baseline Treatment failure (breakthrough or relapse)	• RAS selection

HCV, hepatitis C virus; NGS, next-generation sequencing; RAS, resistance-associated substitution; SVR, sustained virological response; TMA, transcription-mediated amplification.

∗ Viral genome detectable on treatment and in sufficient quantity to permit sequencing.

## HEV and other orthohepeviruses

HEV, also known as *Orthohepevirus* A, now a member of the genus *Paslahepevirus*, can establish a chronic infection in immunocompromised patients that can ultimately lead to serious complications, including rapid progression to cirrhosis and liver failure.^83^ There are eight known genotypes of HEV, with HEV-1 through HEV-4 being the most prevalent worldwide.^83^ Ribavirin, used off-label, reduces viral replication and can lead to definitive HEV clearance. In ribavirin non-responders, PegIFNα has been used in the absence of contraindications. However, there is room for new, more specific treatments for chronic HEV infection, and thus for clinical trials to assess the efficacy of such new treatments.

HEV RNA is a component of viral particles and can be detected and quantified in peripheral blood or faeces as a direct marker of HCV replication using real-time quantitative reverse-transcription PCR. Commercial and in-house assays are available. Whether HEV RNA can be reliably detected in whole blood collected on DBS has been poorly studied.^84^ The presence of HEV RNA in blood and/or faeces confirms the diagnosis of chronic infection and indicates the need for therapy. In clinical trials, the HEV genotype must be determined prior to treatment. The undetectability of HEV RNA after the end of treatment is the endpoint of successful therapy, indicating a definitive cure of the infection. In the event of treatment failure, resistance testing is required, depending on the region(s) targeted by the agents used.

Other *Orthohepevirus* species (B, C and D) have been described in various animal species. *Orthohepevirus* C (genus *Rocahepevirus*) has been shown to infect humans in Europe and Asia.^85^^,^^86^ We recently reported the first European case of chronic *Orthohepevirus* C infection associated with cirrhosis in an immunosuppressed patient and showed that HEV RNA assays are unable to diagnose *Orthohepevirus C* infections.^87^ Specific assays will need to be developed if chronic non-A *Orthohepevirus* infections require clinical trials in the future.

## Conclusions

Chronic hepatitis virus infections remain a major public health problem with nearly 300 million people infected worldwide. Progress has been made in developing effective antiviral strategies, but there is still a need to develop new hepatitis drugs and conduct clinical trials to assess their efficacy and safety. Virological markers remain the cornerstone of efficacy assessment in clinical trials, provided that the right assays are used according to a strict, standardised schedule. Ultimately, the gold standard endpoint of any therapeutic development is clinical benefit to the patient.

## Abbreviations

ALT, alanine aminotransferase; cccDNA, covalently closed circular DNA; DAA, direct-acting antivirals; DBS, dried blood spot; HBcrAg, hepatitis B core-related antigen; HBeAg, hepatitis B e antigen; HBsAg, hepatitis B surface antigen; HBV, hepatitis B virus; HCV, hepatitis C virus; HDV, hepatitis D virus; HEV, hepatitis E virus; LLOQ, lower limit of quantitation; LOD, limit of detection; NGS, next-generation sequencing; PegIFNα, pegylated interferon-α; RASs, resistance-associated substitutions; RDTs, rapid diagnostic tests; SVR, sustained virological response; TMA, transcription-mediated amplification; WHO, World Health Organization.

## Financial support

The author did not receive any financial support to produce this manuscript.

## Conflict of interest

The author has served as an advisor and/or speaker for Abbott, Abbvie, Gilead and GSK.

Please refer to the accompanying ICMJE disclosure forms for further details.

## Declaration of Generative AI and AI-assisted technologies in the writing process

During the preparation of this work, the author used DeepL Write in order to improve English syntax. After using this tool/service, the author reviewed and edited the content as needed and takes full responsibility for the content of the publication.
